# TIminer: NGS data mining pipeline for cancer immunology and immunotherapy

**DOI:** 10.1093/bioinformatics/btx377

**Published:** 2017-06-15

**Authors:** Elias Tappeiner, Francesca Finotello, Pornpimol Charoentong, Clemens Mayer, Dietmar Rieder, Zlatko Trajanoski

**Affiliations:** Division of Bioinformatics, Biocenter, Medical University of Innsbruck, Innsbruck, Austria

## Abstract

**Summary:**

Recently, a number of powerful computational tools for dissecting tumor-immune cell interactions from next-generation sequencing data have been developed. However, the assembly of analytical pipelines and execution of multi-step workflows are laborious and involve a large number of intermediate steps with many dependencies and parameter settings. Here we present TIminer, an easy-to-use computational pipeline for mining tumor-immune cell interactions from next-generation sequencing data. TIminer enables integrative immunogenomic analyses, including: human leukocyte antigens typing, neoantigen prediction, characterization of immune infiltrates and quantification of tumor immunogenicity.

**Availability and implementation:**

TIminer is freely available at http://icbi.i-med.ac.at/software/timiner/timiner.shtml.

**Supplementary information:**

Supplementary data are available at *Bioinformatics* online.

## 1 Introduction

Recent breakthroughs in cancer immunotherapy and decreasing costs of next-generation sequencing (NGS) technologies sparked intensive research into tumor-immune cell interactions using genomic tools. The wealth of the generated data and the added complexity pose considerable challenges and require computational tools to process, analyze and visualize the data. Recently, several tools and analytical pipelines have been developed and used to effectively mine tumor immunologic and genomic data and extract information relevant for cancer immunology and immunotherapy that includes: human leukocyte antigens (HLAs) typing, prediction of neoantigens (non-self tumor antigens predicted from somatic mutations binding specific HLA types), tumor-infiltrating immune cells estimated from RNA-sequencing (RNA-seq) data, and expression levels of key molecules like immune checkpoints (e.g. cytotoxic T-lymphocyte-associated antigen 4 (CTLA4) and programmed cell death protein 1 (PD1) and major histocompatibility complex molecules (see our recent review (Hackl *et al.*, 2016)). However, to the best of our knowledge, there is currently no easy-to-use analytical pipeline to perform integrative immunogenomic analyses. Several tools have been recently published but provide only limited functionality like the identification of tumor neoantigens (Supplementary Table S1). Moreover, assembly of such analytical pipelines and execution of multi-step workflows are laborious and involves a large number of intermediate steps with many dependencies and parameter settings.

We therefore developed TIminer (Tumor Immunology miner), an analytical pipeline to perform integrative immunogenomic analyses using NGS data that is easy to install and use. TIminer integrates state-of-the-art bioinformatics tools to analyze single-sample RNA-seq data and somatic DNA mutations to characterize the tumor-immune interface including: (i) genotyping of HLAs from NGS data, (ii) prediction of tumor neoantigens using mutation data and HLA types, (iii) characterization of tumor-infiltrating immune cells from bulk RNA-seq data; and (iv) quantification of tumor immunogenicity from expression data.

## 2 Tool description

**Fig. 1:**
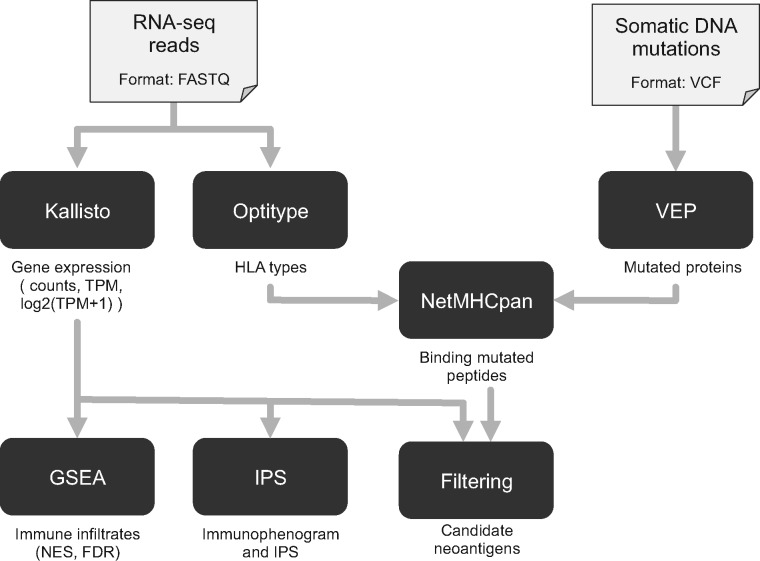
The scheme illustrates the different computational tools integrated in TIminer, the input/output data, and the data flow between the tools

TIminer considers RNA-seq reads and somatic DNA mutations to perform immunogenomic analyses (Fig. 1). RNA-seq reads must be provided as FASTQ files, whereas files of somatic DNA mutations should follow the Variant Call Format. The different analyses, together with their input and output data, are described in the following.

- RNA-seq FASTQ files are used to quantify gene expression with Kallisto (Bray *et al.*, 2016) as: gene-specific counts, transcripts per millions (TPM), and normalized log_2_(TPM+1).
- FASTQ files are analyzed with Optitype (Szolek *et al.*, 2014) to predict class-I HLA types. The output reports the four-digit predictions of the HLA-A, HLA-B and HLA-C alleles.
- Normalized log_2_(TPM+1) are used to estimate the enrichment of tumor-infiltrating immune cell types through gene set enrichment analysis (Subramanian *et al.*, 2005) selecting between three different gene-set schemes (Angelova *et al.*, 2015; Aran *et al.*, 2017; Charoentong *et al.*, 2017) or providing a file with custom gene sets. The output files report the normalized enrichment scores and the associated false discovery rate (FDR)-corrected *P*-values.
- Normalized log_2_(TPM+1) are also used to depict the major determinants of tumor immunogenicity through an immonophenogram and to compute the immunophenoscore, representing the overall tumor immunogenicity (Charoentong *et al.*, 2017).
- Single-nucleotide DNA mutations affecting coding regions are annotated by VEP (McLaren *et al.*, 2016) and used to predict the sequences of the affected proteins.
- The mutated proteins arising from missense mutations, together with the predicted class-I HLA types, are subjected to NetMHCpan (Nielsen and Andreatta, 2016) to predict mutated peptides binding to HLA molecules.
- Binding peptides are filtered considering TPM to select only candidate neoantigens arising from expressed genes. Alternatively, sensitive filtering of neoantigens can be performed considering allele-specific gene expression (see Supplementary Material).

## 3 Implementation

The computational tools performing the immunogenomic analyses integrated in the pipeline have different dependencies, interfaces, and input/output data formats. The installation and assembly of such tools is very laborious and time consuming. TIminer was explicitly designed and implemented to overcome these drawbacks.

The software was pre-built into a ready-to-use Docker image to speed up and simplify the installation process, thereby preventing possible dependency issues and version conflicts. The TIminer framework can be installed on Unix operating systems, including Mac OS, using a one-click installer. In order to simplify the access to the individual tools, we provide a unified Python application programming interface (API). The TIminerAPI wraps the tool calls into the Docker image, introduces parallel execution traces to optimize the computation, and handles all required data pre- and post-processing steps. The single tools are accessible over the TIminerAPI individually or can be run together as part of the full pipeline, available as a python script. In addition, TIminer encloses a graphical user interface that enables single-patient data analysis on standard desktop computer.

Given the size of NGS input data, we recommend analysis of large cohorts of patients with TIminer on computing cluster units, which are available in many institutions. Single-patient data can be analyzed on desktop computers or laptops. Example files, provided within the TIminer package, can be used to test the full pipeline (see Supplementary Material).

## 4 Conclusions

We present here TIminer, the first analytical pipeline that performs integrative immunogenomic analyses from NGS data including HLA typing, neoantigen prediction, characterization of immune infiltrates, and quantification of tumor immunogenicity. Moreover, the pre-built and ready-to-use Docker image enables simple installation procedure. Hence, TIminer represents a valuable tool for basic and translational research in cancer immunology and can expedite the development of precision immuno-oncology. Although developed for cancer immunology and immunotherapy, TIminer provides the means to study also autoimmune, inflammatory, or infectious diseases.

## Funding

This work was supported by the Austrian Science Fund (FWF): W1101-B18 (DK Molecular Cell Biology and Oncology), the European Union's Horizon 2020 research and innovation program under grant agreement No. 633592 (project APERIM: Advanced Bioinformatics Tools for Personalised Cancer Immunotherapy), and the Austrian National Bank (Jubiläumsfondsprojekt No. 16534).

*Conflict of Interest*: none declared.

## Supplementary Material

Supplementary Material
