# Supplementary material for: TIminer: NGS data mining pipeline for cancer immunology and immunotherapy
Source: Bioinformatics. 2017 Jun 15;33(19):3140–1. doi: 10.1093/bioinformatics/btx377 (PMC5870678; doi:10.1093/bioinformatics/btx377)
Supplement: Supplementary Material [file supplementary_material_btx377.pdf]

Supplementary Note:

Quick-start: running TIminer pipeline on mock example files

After installation, the full pipeline can be easily run on mock example files provided together with the TIminer package.

The pipeline can be run from the TIminer 'script' directory by executing the following command:

```
python TIminerPipeline.py --input ../samples/inputInfo.txt --out
../samples/out
```

Alternatively, the mock example files can be analyzed through a graphical user interface (GUI). The GUI can be started by executing from the TIminer 'script' directory the following command:

```
python TIminerUI.py
```

Once the GUI is started (Supplementary Figure S1), the pipeline can be run on the examples files through three simple steps:

1. Click the "Load Examples" button;
2. Specify the path to the "Output directory";
3. Click the "Run" button.

The TIminer pipeline generates several output files. We refer the reader to TIminer online documentation for a full description of the output files (http://icbi.i-med.ac.at/software/timiner/doc/index.html#output-files), and discuss in the following the interpretation of the major results obtained from the mock examples that describe tumor-immune cell interactions.

- ***HLA typing.*** Optitype predicted heterozygous HLA alleles for the HLA-A gene (HLA-A31:01 and HLA-A26:01) and homozygous alleles for the HLA-B and HLA-C genes (HLA-B38:01 and HLA-C12:03, respectively).

- ***Gene set enrichment.*** From Gene Set Enrichment Analysis (GSEA) results we can see that activated CD4+ T cells (ACT_CD4) are significantly enriched at a false-discovery rate of 5% (NES=2.99, q-value=0.00<0.05). Contrariwise, the enrichment of effector memory CD8+ T cells (TEM_CD8) is not significant (NES=1.35, q-value=0.19>0.05). Activated B cells (ACT_B_CELL) are instead depleted, as their NES score is negative (NES=-2.32, q-value=0.01).

- ***Immunophenoscore.*** From the immunophenoscore plot (Supplementary Figure S2) we can see that HLA-related genes are strongly up-regulated (top-left outer sector, in dark red), while genes related to both effector immune cells (EC, top-right sector) and suppressive immune cells (SC, bottom-right sector) are slightly up-regulated. Most of the checkpoints molecules (CP) with immunoinhibitory effects (identified by the "-" sign) are down-regulated, while co-stimulators ("+" sign) are both up- (CD27) and down-regulated (ICOS). Taken all together, these positive and negative contributions can be summarized in an immunophenoscore of 10 (on a [0-10] scale), representing a good immunophenotype, i.e. a tumor which is likely to elicit an effective immune response.

- ***Neoantigens.*** The expressed neoantigens consist of seven mutated peptides arising from two genes: NCOA6 and TP53. The original pool of mutated peptides comprised eight peptides from three genes, but one of them was not expressed (TP53TG3D). Among the expressed neoantigens, the MNRRPILTI peptide, arising from an R>G missense mutation in TP53, was predicted to bind to HLA-C12:03 with high affinity (407.5 nM), whereas a lower affinity was predicted for its wild-type version MNRGPILTI (1059.7 nM).

Advanced filtering

The TIminer function `filterNeoantigenDir` (or `filterNeoantigenFile` for single-subject analysis) selects neoantigens that arise from expressed genes, which are identified from the files of transcripts-per-millions (TPM) generated by Kallisto (Bray et al., 2016) (see Figure 1 in the main text). Alternatively, an advanced filtering scheme implemented in the `sensitiveFilterNeoantigenDir` function (or `sensitiveFilterNeoantigenFile` for single-subject analysis) can be selected (Supplementary Figure S3). This function performs sensitive mapping of the RNA-seq reads with HiSat2 (Kim et al., 2015) and then calculates the coverage of each mutation from with ASEReadCounts function from the Genome Analysis Toolkit (McKenna et al., 2010). Finally, from the list of binding mutated peptides identified by NetMHCpan (Nielsen and Andreatta, 2016), those arising from mutations with an RNA-seq read coverage ≥5 are selected (default value which can be modified with the `countThresh` parameter).

The sensitive filtering can be activated in the TIminer pipeline by specifying the `--sensitiveFiltering` parameter:

```
python TIminerPipeline.py --input INPUT --out OUT --sensitiveFiltering
```

Please note that this option, with respect to the default filtering scheme, is more computationally demanding.

Supplementary Figure S1. TIminer graphical user interface (GUI).

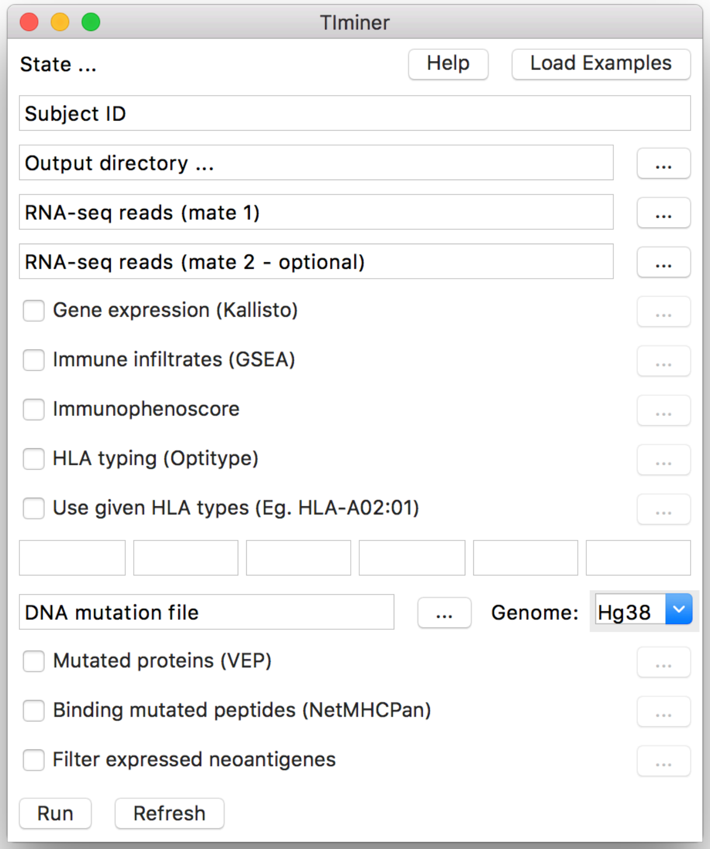

Supplementary Figure S2. The immunophenogram obtained from the mock example data. The outer sectors represent the z-scored expression of the genes or immune cell types determining tumor immunogenicity, together with their weight (either positive “+” or negative “-“); gene z-scores are averaged for cell types. The immune cell types are: activated CD4^{+} or CD8^{+} T cells (Act CD4 or Act CD8), effector memory CD4^{+} or CD8^{+} T cells (Tem CD4 or Tem CD8), central memory CD4^{+} or CD8^{+} T cells (Tcm CD4 or Tcm CD8), regulatory CD4+ T cells (Treg), and myeloid-derived suppressor cells (MDSC). The inner sectors summarize the z-scores into four scores, one for each major determinant of tumor immunogenicity: genes related to antigen processing and presentation (MHC), checkpoints molecules and immunomodulators (CP), effector T-cells (EC), and suppressive immune cells (SC). The immunophenoscore (IPS) is an aggregated score representing the overall tumor immunogenicity on an arbitrary scale from 0 to 10.

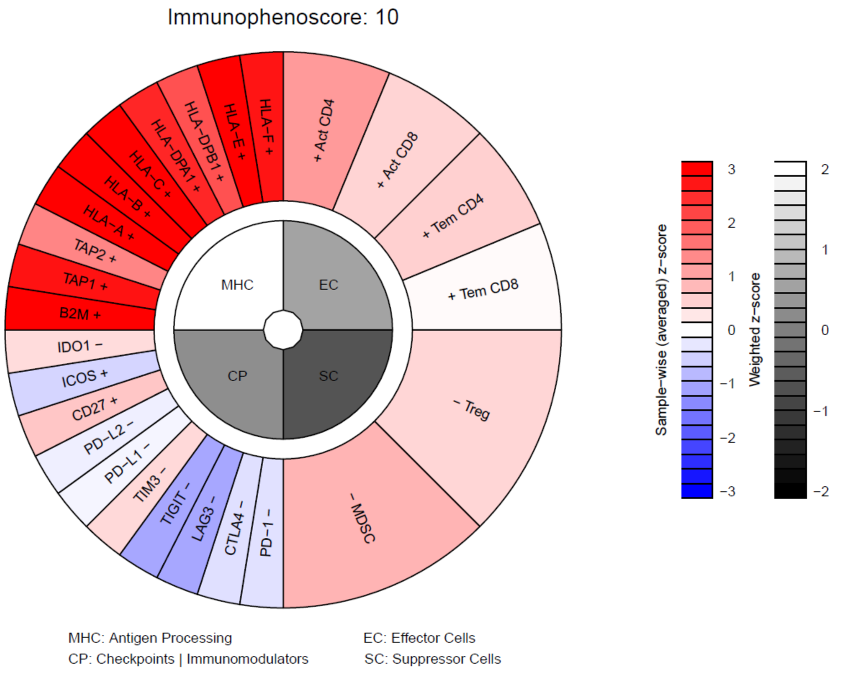

Supplementary Figure S3. Scheme of the TIminer pipeline with sensitive filtering of neoantigens, which filters the list of binding mutated peptides identified by NetMHCpan (Nielsen and Andreatta, 2016) considering only the expressed mutations. Expressed mutations are identified through a two-step procedure: (i) sensitive re-alignment of RNA-seq reads with HiSat2 (Kim et al., 2015); and (ii) computation of RNA-seq-read coverage of the somatic mutations with the Genome Analysis Toolkit (GATK)(McKenna et al., 2010).

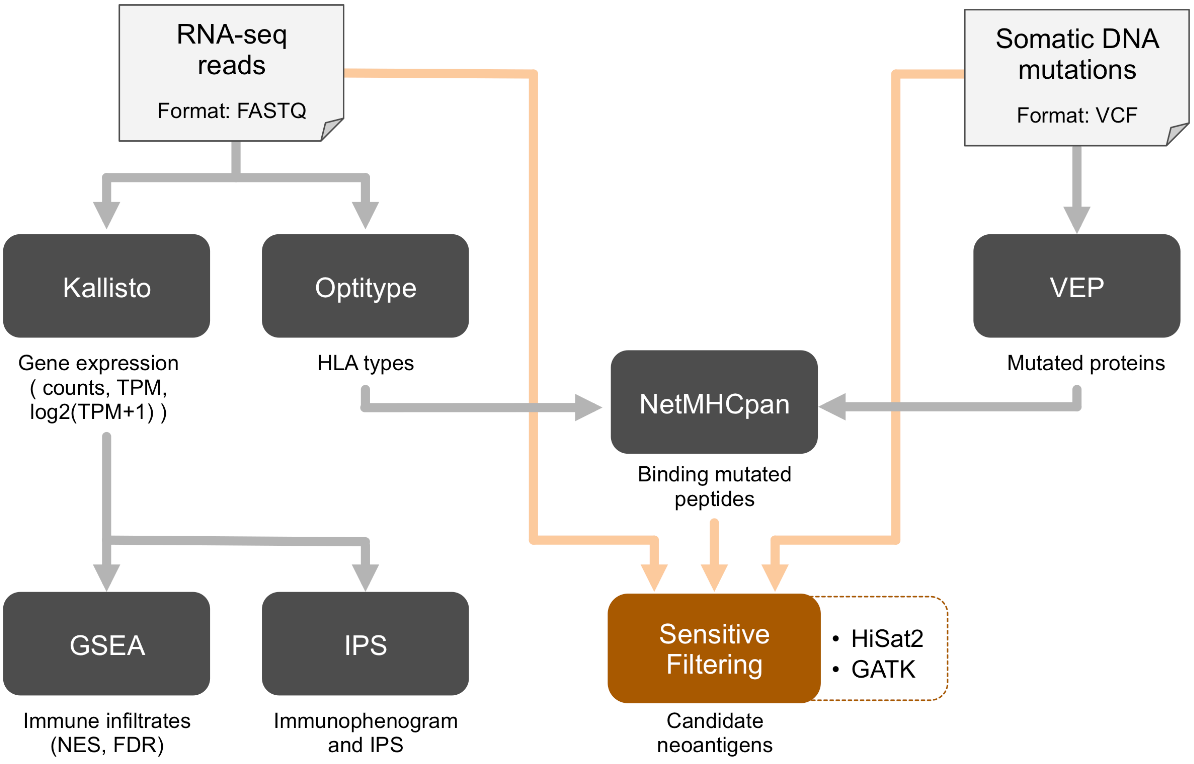

Supplementary Table S1. Standalone, computational pipelines for class-I neoantigen prediction available in the literature.

Method	Predictions	URL	Ref
FRED 2	Mutated peptide (from SNPs and indels), HLA typing, proteasomal cleavage, TAP transport, peptide-HLA binding affinity, peptide prioritization, and vaccine design	http://fred-2.github.io	(Schubert et al., 2016)
INTEGRATE-neo	HLA typing, mutated peptide (from gene fusions), peptide-HLA binding affinity	https://github.com/ChrisMaherLab/INTEGRATE-Neo	(Zhang et al., 2017)
MuPeXI	Mutated peptide (from SNPs, frameshift mutations, and indels), peptide-HLA binding affinity, peptide prioritization considering also gene expression, allele frequency, and protein self-dissimilarity	http://www.cbs.dtu.dk/services/MuPeXI/	(Bjerregaard et al., 2017)
NetCTL	Proteasomal cleavage, TAP transport, peptide-HLA binding affinity, and combined score for peptide prioritization	http://www.cbs.dtu.dk/services/NetCTL	(Larsen et al., 2007)
NetEpi	Peptide-HLA binding affinity and stability, T-cell propensity, and combined score for peptide prioritization	http://www.cbs.dtu.dk/services/NetTepi	(Trolle and Nielsen, 2014)
pVAC-seq	Mutated peptide (from SNPs), peptide-HLA binding affinity, and peptide prioritization considering also NGS read coverage and gene expression	http://github.com/griffithlab/pVAC-Seq	(Hundal et al., 2016)

HLA: human leukocyte antigen; indel: insertion or deletion; NGS: next-generation sequencing; SNP: single-nucleotide polymorphism; TAP: transporter associated with antigen processing.

Supplementary References

Bjerregaard, A.-M., Nielsen, M., Hadrup, S.R., Szallasi, Z., and Eklund, A.C. (2017). MuPeXI: prediction of neo-epitopes from tumor sequencing data. Cancer Immunol. Immunother. CII.

Bray, N.L., Pimentel, H., Melsted, P., and Pachter, L. (2016). Near-optimal probabilistic RNA-seq quantification. Nat. Biotechnol. *34*, 525–527.

Hundal, J., Carreno, B.M., Petti, A.A., Linette, G.P., Griffith, O.L., Mardis, E.R., and Griffith, M. (2016). pVAC-Seq: A genome-guided in silico approach to identifying tumor neoantigens. Genome Med. *8*, 11.

Kim, D., Langmead, B., and Salzberg, S.L. (2015). HISAT: a fast spliced aligner with low memory requirements. Nat. Methods *12*, 357–360.

Larsen, M.V., Lundegaard, C., Lamberth, K., Buus, S., Lund, O., and Nielsen, M. (2007). Large-scale validation of methods for cytotoxic T-lymphocyte epitope prediction. BMC Bioinformatics *8*, 424.

McKenna, A., Hanna, M., Banks, E., Sivachenko, A., Cibulskis, K., Kernytsky, A., Garimella, K., Altshuler, D., Gabriel, S., Daly, M., et al. (2010). The Genome Analysis Toolkit: a MapReduce framework for analyzing next-generation DNA sequencing data. Genome Res. *20*, 1297–1303.

Nielsen, M., and Andreatta, M. (2016). NetMHCpan-3.0; improved prediction of binding to MHC class I molecules integrating information from multiple receptor and peptide length datasets. Genome Med. *8*, 33.

Schubert, B., Walzer, M., Brachvogel, H.-P., Szolek, A., Mohr, C., and Kohlbacher, O. (2016). FRED 2: an immunoinformatics framework for Python. Bioinforma. Oxf. Engl. *32*, 2044–2046.

Trolle, T., and Nielsen, M. (2014). NetTepi: an integrated method for the prediction of T cell epitopes. Immunogenetics *66*, 449–456.

Zhang, J., Mardis, E.R., and Maher, C.A. (2017). INTEGRATE-neo: a pipeline for personalized gene fusion neoantigen discovery. Bioinforma. Oxf. Engl. *33*, 555–557.
